# MALAT1 regulates miR-34a expression in melanoma cells

**DOI:** 10.1038/s41419-019-1620-3

**Published:** 2019-05-17

**Authors:** Fei Li, Xinji Li, Li Qiao, Wen Liu, Chengshan Xu, Xiaogang Wang

**Affiliations:** 1 Department of Dermatology, Air Force Medical Center, PLA, Beijing, China; 2 Department of Radiation Oncology, Air Force Medical Center, PLA, Beijing, China; 3 National Center for Clinical Laboratories, Beijing Hospital, National Center of Gerontology, Beijing, China; 4 Beijing Advanced Innovation Center for Big Data-based Precision Medicine, Beihang University, Beijing, China

**Keywords:** Medical research, Genetics research

## Abstract

Melanoma is one of the most common skin malignancies. Both microRNAs and long non-coding RNAs (lncRNAs) have critical roles in the progression of cancers, including melanoma. However, the underlying molecular mechanism has not been fully characterized. We demonstrated that miR-34a is negatively correlated with MALAT1 in melanoma cells and tumor specimens. Interestingly, MALAT1, which contains functional sequence-specific miR-34a-binding sites, regulates miR-34a stability in melanoma cells and in vivo. Importantly, MALAT1 was significantly enriched in the Ago2 complex, but not when the MALAT1-binding site of miR-34a was mutated. Furthermore, MALAT1 could be shown to regulate *c-Myc* and *Met* expression by functioning as a miR-34a sponge. Our results reveal an unexpected mode of action for MALAT1 as an important regulator of miR-34a.

## Introduction

Melanoma is an aggressive cancer involving pigment-containing cells known as melanocytes that are found predominantly in the skin. Melanoma is the leading cause of skin cancer death in the United States, with an estimated 87,110 new cases and 9730 deaths in 2017^1^. In China, it is estimated that there were 8000 new cases of melanoma and 3200 deaths due to this disease in 2015^2^. The molecular mechanisms of melanoma need to be further studied, and the identification of molecular drivers may enable the development of novel melanoma therapies.

Recent studies have revealed that non-coding RNAs, including microRNAs (miRNAs) and long non-coding RNAs (lncRNAs), play important roles in various pathophysiological processes, and are frequently dysregulated in many types of cancer^3–9^. Several studies have demonstrated that miRNAs, which regulate target mRNAs at the post-transcriptional level, are involved in the pathogenesis of all tumor types. In addition, lncRNAs comprising non-protein-coding transcripts longer than 200 nucleotides contribute to diverse biological functions in human diseases, including cancer^10–13^.

Metastasis-associated lung adenocarcinoma transcript 1 (MALAT1), is one of the most abundant and evolutionarily conserved lncRNAs. The deregulation and function of MALAT1 have been established in several solid tumors, including bladder cancer, lung cancer, colorectal cancer, esophageal squamous cell carcinoma, melanoma, breast cancer, and hepatocellular carcinoma^14–20^. MALAT1 levels were markedly higher in melanomas than in paired adjacent normal tissues^21^. MALAT1 was also identified as a competing endogenous RNA (i.e., miR-183 sponge), and thus regulates the molecular expression of *ITGB1* in melanoma^22^. Furthermore, MALAT1 promotes melanoma cell growth and invasion by silencing miR-140 or miR-22^17,23^.

In an earlier investigation, miR-34a was characterized as a tumor suppressor, in part, via the SIRT1-p53 pathway^24^. Therefore, miR-34 dysregulation may be involved in the development of some cancers^25,26^. The *c-Myc* gene is often constitutively expressed in malignant tumors, and the encoded protein is believed to regulate the expression of 15% of all genes in the human genome^27^. Some of the regulated genes are involved in cell proliferation, metastasis, and apoptosis, thereby contributing to cancer development^28–31^. Meanwhile, Met is a receptor tyrosine kinase family member encoded by the proto-oncogene *Met*. Abnormal activation of Met in cancer is correlated with poor prognosis, likely because aberrantly active Met triggers tumor growth, angiogenesis, and metastasis^32–35^. In addition, Met is deregulated in many types of human malignancies, including breast cancer, lung cancer, bladder cancer, hepatocellular carcinoma, and melanoma^36–40^. To date, whether MALAT1 affects *c-Myc* and *Met* expression levels via a competing endogenous RNA of miR-34a remains unclear.

In this study, we investigated the MALAT1 and miR-34a levels in melanoma, and explored the correlation between MALAT1 and miR-34a production. Importantly, we identified miR-34a as a target of MALAT1, the latter of which contains functional sequence-specific miR-34a-binding sites. These findings imply that MALAT1 helps regulate miR-34a expression, thereby expanding the functions of MALAT1 to include post-transcriptional regulatory activities.

## Materials and methods

### Cell culture

The melanoma cell line A375 was purchased from the National Infrastructure of Cell Line Resource (Cell Resource Center of the Chinese Academy of Medical Sciences, Beijing, China) and cultured in DMEM supplemented with 2 mM l-glutamine and 10% FBS. Cells were grown at 37 °C under humid conditions with 5% CO_2_.

### Tissue samples

Tissues were obtained from patients who were diagnosed with melanoma and treated between Mar 2015 and Feb 2017 at the Department of Dermatology, Air Force Hospital, People’s Liberation Army. Skin tissues were collected from 20 patients with melanocytic nevi (matched by sex and age) as controls. Every patient involved in the study provided written informed consent that was approved by the Ethics Committee of the Air Force Hospital. All signed consent forms were saved by the Ethics Committee. The tissue samples were frozen within 30 min of surgery and stored in liquid nitrogen until use. Tissue specimens were cut into blocks (3–4 mm thick) and then fixed in fresh 10% neutral-buffered formalin for 16–32 h at room temperature (25 °C) before being embedded in paraffin for a subsequent RNA scope analysis.

### Oligonucleotides, plasmids, and transfection

The following miRNAs were synthesized by Integrated Biotech Solutions (Shanghai, China): miR-NC, miR-34a mimics, miR-34a-mut, anti-miR-34a, anti-miR-34a-mut, biotin-miR-NC, biotin-miR-34a-mut, and biotin-miR-34a. Control siRNA and MALAT1 siRNA were purchased from Bioneer (Shanghai, China). The full-length 3′ untranslated regions (3′-UTR) of *c-Myc*, *c-Met*, wt-MALAT1, and mut-MALAT1 were subcloned into the psiCHECK-2 plasmid. For mismatch constructs, seven mismatches, which are indicated in bold letters in the following MALAT1 sequence: **ACCGUCA**GACGGGAGUUUUCGA (Fig. 4), were introduced into the putative target site, which was modified to **UGGCAGU**GACGGGAGUUUUCGA. For the transfections involving DNA plasmids and oligonucleotides, Lipofectamine 2000 (Life Technologies Corporation, Grand Island, NY) was used according to the manufacturer’s instructions.

### RNA sequencing

The A375 cells were transfected with MALAT1 siRNA, after which total RNA was isolated using the TRIzol reagent. The A375 cells transfected with control siRNA were used as the control. Each cell line was analyzed with three biological replicates. RNA-sequencing and data analysis were performed by Integrated Biotech Solutions.

### RNA isolation, reverse transcription, and quantitative real-time polymerase chain reaction analysis

Total RNA was isolated from cells or tissues using a TRIzol kit (Invitrogen, Carlsbad, CA) following the manufacturer’s instructions. The extracted RNA was used as the template for a reverse transcription with the SuperScript III Reverse Transcriptase (Invitrogen). The miR-34a and MALAT1 expression levels were analyzed in a quantitative real-time polymerase chain reaction (qRT-PCR) assay, which was performed with the 7500 Fast Real-Time PCR System (Applied Biosystems, Foster City, CA, USA) according to the manufacturer’s instructions. The U6 small nuclear RNA and *GAPDH* gene were used as internal controls for analyzing the miRNA and mRNA levels, respectively. The following primers were designed for the qRT-PCR assay: MALAT1 forward 5′-TCCAGAAAGAGGGAGTTG-3′, reverse 5′-GAAGCCAGACCCAGTAAG-3′; *GAPDH* forward 5′-CCATGCCATCACTGCCACCC-3′, reverse 5′-GCCAGTGAGCTTCCCGTTCAG-3′; and miR-34a-5p forward 5′-TGGCAGTGTCTTAGCTGGTTGT-3′, reverse 5′-CTCAACTGGTGTCGTGGAGTC-3′. The resulting qRT-PCR data were analyzed using the 2^−ΔΔCt^ method. All reactions were run in triplicate.

### Biotin pull-down assay

A biotinylated-miR-34a-capture assay was carried out as previously described^41^. Briefly, biotin-miR-NC, biotin-miR-34a-mut, and biotin-miR-34a were separately transfected into A375 cells. At 48 h after transfection, cells were lysed and the resulting lysate was added to 30 μL beads (Dynabeads MyOne Streptavidin C1, Life Technologies). After agitating the lysate-bead mixture on a rotary shaker for 4 h at 4 °C, RNA was extracted from the beads with TRIzol Reagent (Life Technologies) and analyzed in a qRT-PCR assay.

### Western blot and antibodies

Treated A375 cells were harvested and lysed in protein lysis buffer (50 mM Tris-HCl, 150 mM NaCl, 0.1% NP-40, 5 mM EDTA, and 10% glycerol) supplemented with a protease inhibitor cocktail (Sigma-Aldrich, St. Louis, MO USA). Protein concentration was determined with the BCA Protein Assay Kit (P0011, Beyotime, Shanghai, China). Proteins were separated by 12% or 9% SDS-PAGE and transferred to a PVDF membrane (IPVH00010, Millipore, Billerica, MA, USA) and immunoblotted with the following antibodies: anti-Met (ab51067, Abcam, Cambridge, MA, USA), anti-c-Myc (Abcam, ab32072), and anti-β-actin (Sigma, A5316).

### Luciferase assay

Lipofectamine 2000 (Invitrogen) was used to co-transfect A375 cells with psiCHECK-2, psiCHECK-2-c-Myc, psiCHECK-2-Met, MALAT1 siRNA, and anti-miR-34a or anti-miR-34a-mut according to the manufacturer’s instructions. Three independent transfection experiments were conducted, each with three technical replicates. In all experiments, the firefly luciferase gene in psiCHECK-2 was used as a control to normalize the transfection efficiency. At 48 h after transfection, the firefly and Renilla luciferase activities were quantified with the Dual-Luciferase Reporter Assay System (Promega) and the BMG Labtech microplate reader.

### Lentivirus production and stable cell lines

The MALAT1 and MALAT1-mut sequences were ligated into separate pLVX-IRES-Puro vectors to construct the MALAT1 and MALAT1-mut overexpression plasmids. The HEK293T cells were co-transfected with the pLVX-IRES-Puro-MALAT1, pLVX-IRES-Puro-MALAT1-mut, psPAX2, and pMD2.G plasmids. At 48 h after transfection, the supernatant was collected and injected into nude mice. Meanwhile, a lentiviral small hairpin RNA targeting MALAT1 (sh-lncRNA-MALAT1) and sh-NC (negative control) were designed and cloned into the pLVshRNA-Puro vector according to the manufacturer’s instructions (Inovogen Tech. Co., Beijing, China). The A375 cells were grown to ~40% confluence, after which they were infected with lentiviral particles in complete medium for 48 h and then selected with puromycin.

### Animal experiments

Cells carrying pLVX-IRES-Puro-MALAT1 and pLVX-IRES-Puro-MALAT1-mut, or sh-lncRNA-MALAT1 or sh-NC were injected subcutaneously into the dorsal flanks of 5-week-old male BALB/c nude mice. The xenografts were dissected and total protein and RNA were obtained to analyze MALAT1, miR-34a, c-Myc, and Met levels.

### RNAscope

The MALAT1 expression level was analyzed by Advanced Cell Diagnostics with an RNAscope probe.

### RNA immunoprecipitation and qRT-PCR

An immunoprecipitation experiment involving anti-Ago2 was conducted as previously described^41^. Briefly, A375 cells were harvested at 48 h after transfection with miR-NC, miR-34a mimics, and miR-34a-mut or the MALAT1 expression vector. The cells were lysed and centrifuged at 12,000 × g for 30 min, after which 30 μL anti-FLAG M2 magnetic beads were added to the lysate (Sigma). After agitating the lysate-bead mixture on a rotary shaker for 4 h at 4 °C. The beads were washed three times with washing buffer (50 mM Tris-HCl, 300 mM NaCl, pH 7.4, 1 mM MgCl_2_, and 0.1% NP-40). The pull-down complexes were analyzed by qRT-PCR.

### Statistical analysis

All data are herein presented as the mean ± standard deviation. For all experiments, statistical significance of data was determined by a two-tailed Student’s *t*-test conducted with the SPSS 17.0 program. A *P* value < 0.05 was considered statistically significant.

## Results

### miR-34a is negatively correlated with MALAT1 in melanoma cells

**Fig. 1:**
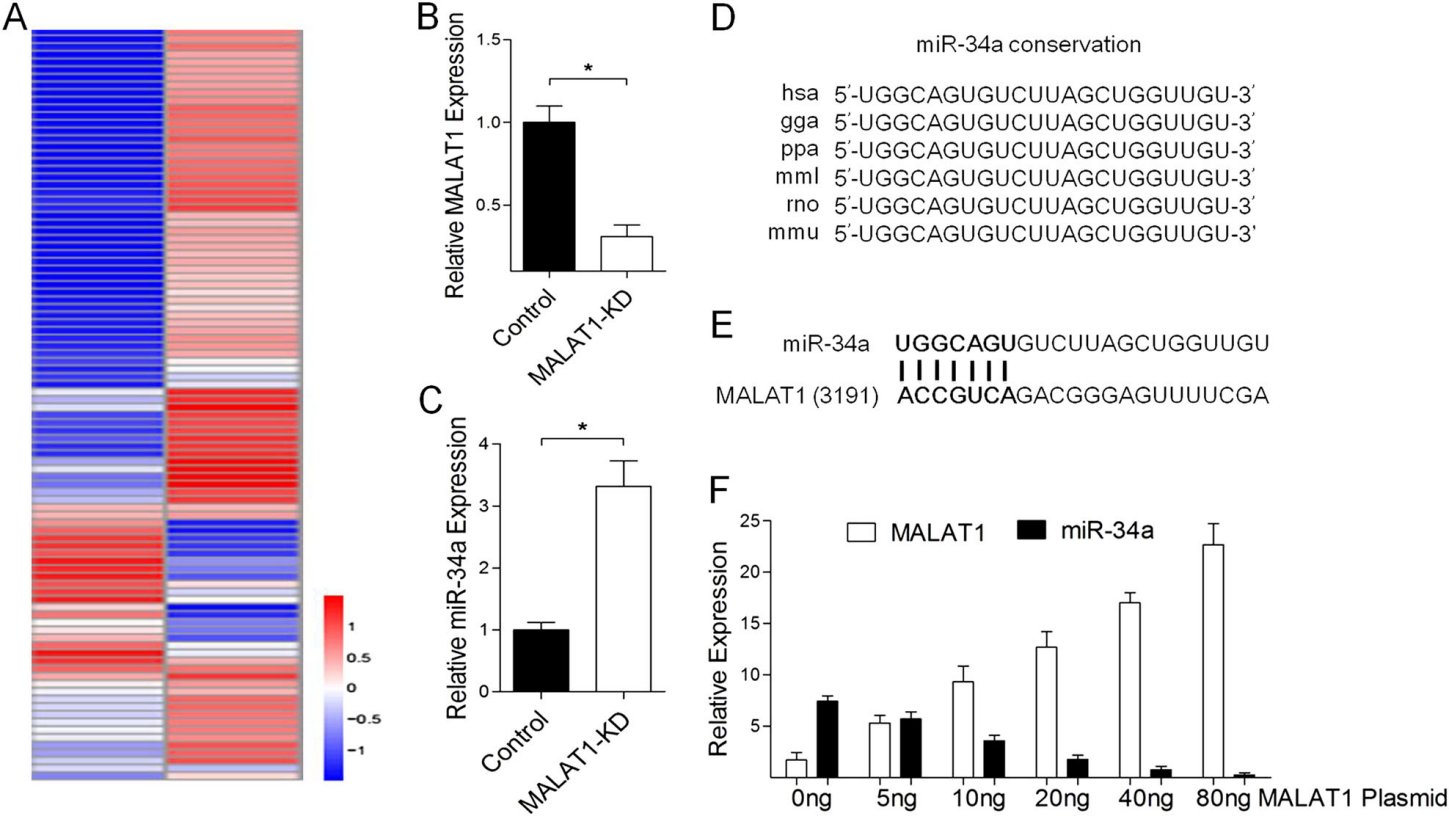
miR-34a is negatively correlated with MALAT1 in melanoma cells. **a** Heat map of differentially expressed miRNAs in A375 cells transfected with the negative control siRNA vector and MALAT1 siRNA (MALAT1-KD). A375 cells were transfected with 20 nM control siRNA or MALAT1 siRNA, and after a 48 h incubation, **b** MALAT1 and **c** miR-34a expression levels were analyzed in a quantitative real-time polymerase chain reaction (qRT-PCR) assay, with the expression data normalized against that of the control. **d** Aligned miR-34a sequences from nine species. **e** Bioinformatics analyses predicted the binding sites between MALAT1 and miR-34a. **f** A375 cells were transfected with different concentrations of MALAT1 expression vectors (0, 5, 10, 20, 40 , and 80 ng), after which miR-34a expression was analyzed in a qRT-PCR assay

There is growing evidence of an important role for MALAT1 in tumorigenesis^42–44^. To investigate the potential mechanisms regulating the effects of MALAT1 on melanoma cells, we analyzed a miRNA-seq transcriptome of A375 melanoma cells transfected with MALAT1 siRNA or a scrambled control. The transcriptome data are presented in Fig. 1a, b. The eight most regulated miRNAs were hsa-miR-34a, hsa-miR-200a-3p, hsa-miR-196a-5p, hsa-miR-107, hsa-miR-196b-5p, hsa-miR-31-3p, hsa-miR-143-3p, and hsa-miR-582-3p. The largest fold change and the most-significant *P* value (*P* *=* 0.00027719) was observed for miR-34a (Table 1). The miR-34a expression levels in MALAT1-knockdown and control A375 cells were validated in a qRT-PCR assay, which indicated that miR-34a expression was consistent with the sequencing data (Fig. 1c). In addition, miR-34a was highly conserved among six species (Fig. 1d). Recent studies have suggested that lncRNAs may function as endogenous RNA sponges that interact with and influence the expression of miRNAs^45,46^. Moreover, a bioinformatics analysis revealed a predicted miR-34a response element in the MALAT1 transcript (Fig. 1e). After a 24h MALAT1 overexpression, we evaluated the miR-34a levels in A375 cells. When MALAT1 expression was enhanced, the miR-34a levels decreased in a dose-dependent manner (Fig. 1f). Thus, MALAT1 appears to negatively regulate miR-34a in A375 cells.

**Table 1:** The eight most changed miRNAs regulated by MALAT1

No.	Reads of KD	Reads of Control	log2.fold_change
hsa-miR-34a	149.893009	75.25562828	0.994061709
hsa-miR-200a-3p	58.4784386	32.17034491	0.862173387
hsa-miR-196a-5p	106.874388	60.31939671	0.825222254
hsa-miR-107	70.5774259	42.51081292	0.731376966
hsa-miR-196b-5p	68.560928	41.36187203	0.729085154
hsa-miR-31-3p	131.744528	85.02162584	0.631841288
hsa-miR-143-3p	118.301209	76.40456917	0.630733995
hsa-miR-582-3p	493.369815	321.1289787	0.619516593

### MALAT1 binds directly to miR-34a in melanoma cells

**Fig. 2:**
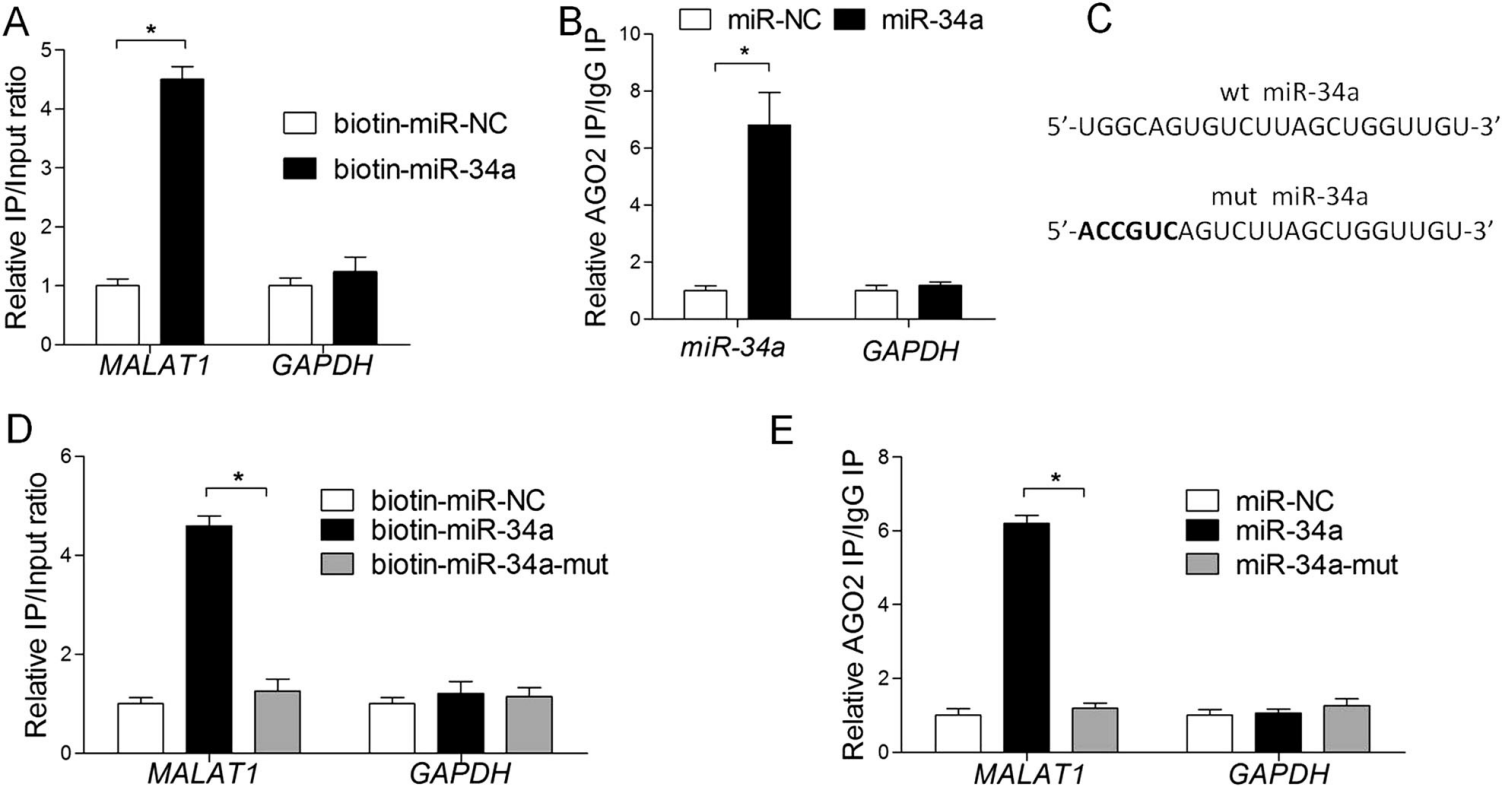
MALAT1 directly binds to miR-34a in melanoma cells. **a** A streptavidin-capture assay was performed for A375 cells transfected with biotin-miR-NC or biotin-miR-34a, followed by a quantitative real-time polymerase chain reaction (qRT-PCR) assay to analyze MALAT1 and GAPDH mRNA levels. **b** An Ago2 immunoprecipitation experiment was completed for A375 cells transfected with control miRNA (miR-NC) or miR-34a, followed by a qRT-PCR assay to analyze the MALAT1 associated with Ago2. **c** Schematic representation of the wild-type miR-34a and mutated miR-34a. **d** Streptavidin -capture assay was performed for A375 cells transfected with biotin-miR-NC, biotin-miR-34a, or mutated biotin-miR-34a, followed by a qRT-PCR assay to analyze MALAT1 and GAPDH mRNA levels. **e** An Ago2 immunoprecipitation experiment was performed for A375 cells transfected with control miRNA (miR-NC), miR-34a, or mutated miR-34a, followed by a qRT-PCR assay to analyze the MALAT1 associated with Ago2

In this study, MALAT1 was detected following the affinity purification of miR-34a-interacting transcripts from A375 cells transfected with biotinylated miR-34a, but not from cells transfected with the biotinylated scrambled control. These results suggest that MALAT1 functions as a miRNA sponge that negatively regulates miR-34a levels. Moreover, neither miR-34a nor the control associated with *GAPDH* mRNA, which was used as a negative control (Fig. 2a). When the MALAT1-binding site of miR-34a was mutated, MALAT1 was not pulled down, indicating that MALAT1 regulated miR-34a in a sequence-specific manner (Fig. 2c, d). Because miRNAs regulate gene expression at post-transcriptional level through the RNA-induced silencing complex (RISC) containing Ago2, an RNA-binding protein immunoprecipitation assay was completed to verify whether miR-34a and MALAT1 are present in the same RISC. We observed that MALAT1 was significantly enriched in the Ago2 complex (Fig. 2b), but not when the MALAT1-binding site of miR-34a was mutated (Fig. 2e). These data indicate that miR-34a binds directly to MALAT1 in A375 cells.

### miR-34a target genes are regulated by MALAT1 in melanoma cells

**Fig. 3:**
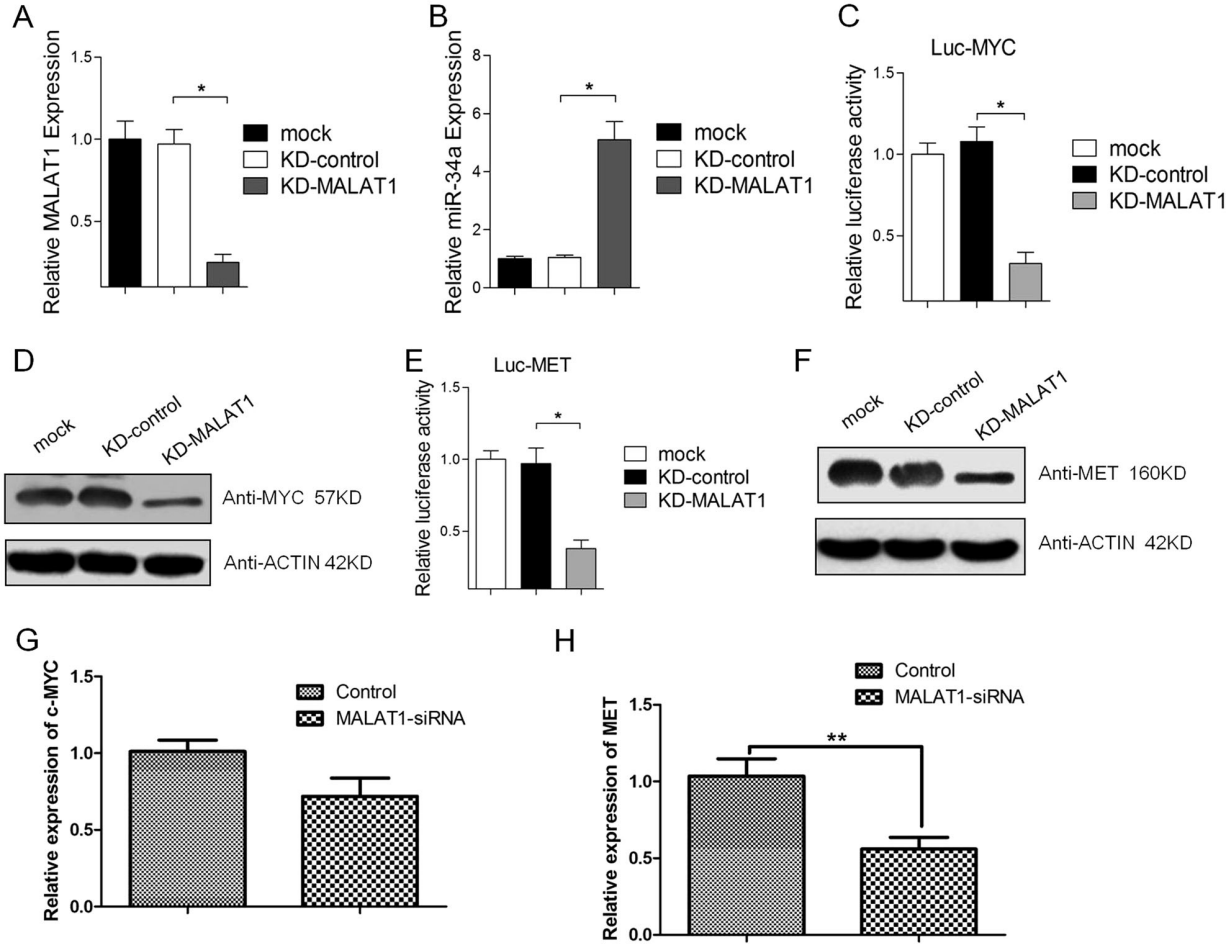
miR-34a target genes are regulated by MALAT1 in melanoma cell. A375 cells were transfected with 20 nM control siRNA or MALAT1 siRNA. After a 48-h incubation, **a** MALAT1 and **b** miR-34a expression levels were analyzed in a quantitative real-time polymerase chain reaction (qRT-PCR) assay, with the expression data normalized against that of the control. After a 72-h incubation post-transfection, the luciferase activities of **c** Luc-c-Myc and **e** Luc-Met were analyzed in a luciferase reporter assay. After a 72-h incubation post-transfection, **d** c-Myc and **f** Met protein levels were analyzed in a western blot, and **g**) *c-Myc* and (**h**) *Met* mRNA levels were analyzed in a qRT-PCR assay

Previous studies confirmed that miR-34a can bind directly to many oncogenes, including *c-Myc* and *Met*, to regulate expression^47–50^. In this study, we used a luciferase reporter assay, qRT-PCR, and a western blot to verify whether c-Myc and Met are regulated by MALAT1. The knockdown of MALAT1 significantly increased the miR-34a level (Fig. 3b). Luciferase reporters containing the *c-Myc* and *Met* 3′-UTR were also constructed, and the knockdown of MALAT1 suppressed the luciferase activity of the *c-Myc* (Fig. 3c) and *Met* (Fig. 3e) reporter vectors. Additional studies showed that the knockdown of MALAT1 may decrease the c-Myc (Fig. 3d) and Met (Fig. 3f) protein levels. Thus, MALAT1 may regulate the expression of the miR-34a target genes *c-Myc* and *Met*. Interestingly, knockdown of MALAT1 suppressed the transcription of *Met* (Fig. 3g) but not *c-Myc* (Fig. 3h).

### MALAT1 functions as a miR-34a sponge in A375 melanoma cells

**Fig. 4:**
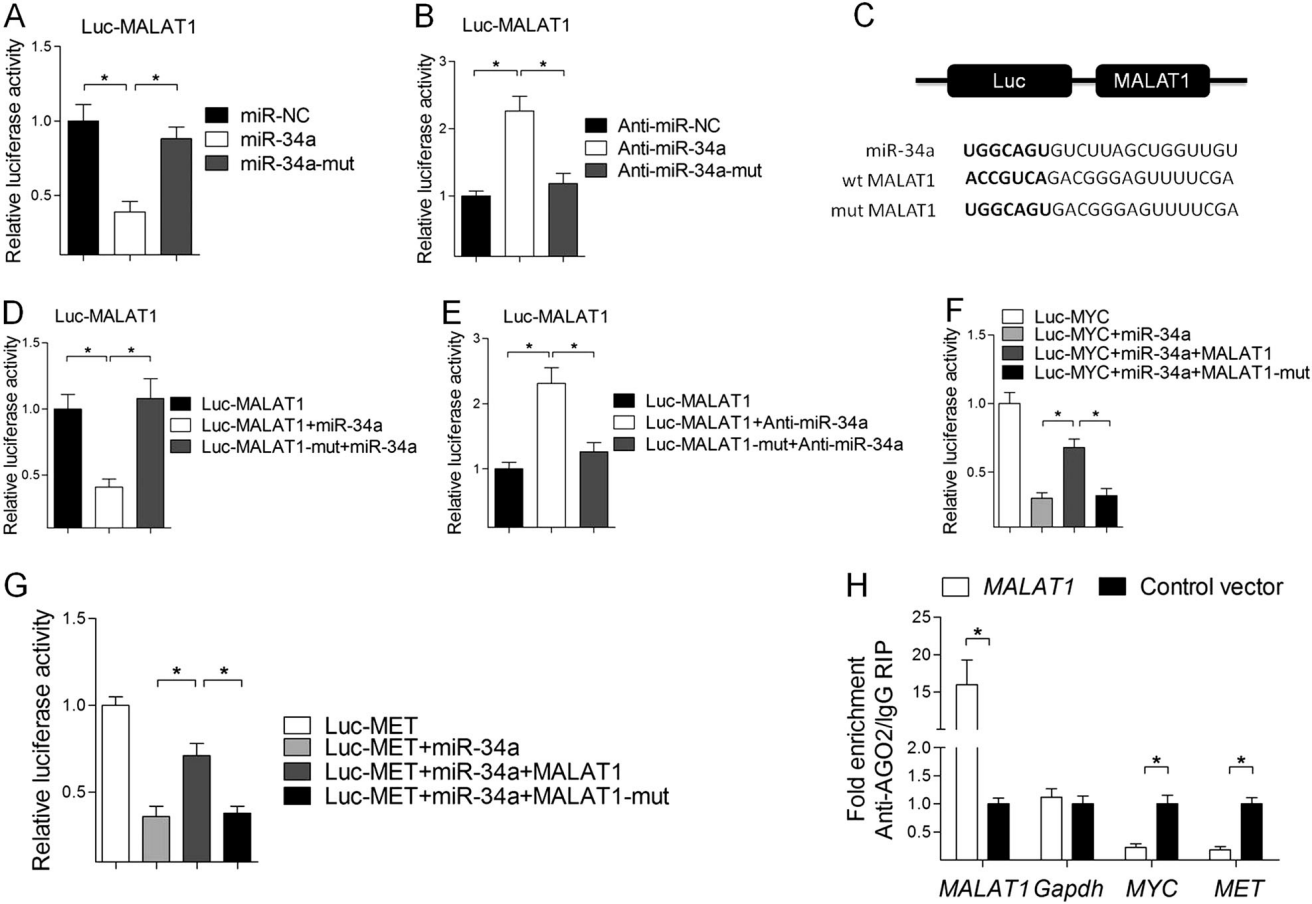
MALAT1 functions as a miR-34a sponge in A375 melanoma cells. **a** Luciferase reporter constructs: Wild-type MALAT1 (wt-MALAT1) and MALAT1 with mutations in the miR-34a-binding sites (Luc-MALAT1-mut) were inserted into the psiCHECK-2 vector. Letters in bold font represent mutation sites. **b** Relative luciferase activity of A375 cells with psiCHECK-2 containing wt-MALAT1 co-transfected with control miRNA (miR-NC), miR-34a, or mutated miR-34a. **c** Relative luciferase activity of A375 cells with psiCHECK-2 containing wt-MALAT1 co-transfected with control miRNA (anti-miR-NC), anti-miR-34a, or mutated miR-34a (anti-miR-34a-mut). **d** Relative luciferase activity of A375 cells with psiCHECK-2 containing wt-MALAT1 and mutated MALAT1 (Luc-MALAT1-mut) co-transfected with miR-34a. **e** Relative luciferase activity of A375 cells with psiCHECK-2 containing wt-MALAT1 and mutated MALAT1 (Luc-MALAT1-mut) co-transfected with anti-miR-34a. **f** Relative luciferase activity of A375 cells with psiCHECK-2 containing c-Myc co-transfected with miR-34a and wt-MALAT1 or mutated MALAT1 (MALAT1-mut) expression vectors. **g** Relative luciferase activity of A375 cells with psiCHECK-2 containing Met co-transfected with miR-34a and wt-MALAT1 or mutated MALAT1 (MALAT1-mut) expression vectors. **h** An Ago2 immunoprecipitation experiment was performed for A375 cells transfected with the control vector or MALAT1 expression vector, followed by a quantitative real-time polymerase chain reaction assay to analyze the MALAT1, GAPDH, c-Myc, and Met associated with Ago2

A Dual-Luciferase Reporter Assay System involving the wild-type (WT) and mutant-type (Mut) MALAT1-binding sites was used to investigate whether miR-34a is targeted by MALAT1. The assay results indicated that the overexpression of miR-34a, but not miR-34a-mut, suppressed the luciferase activity of the WT reporter vector (Fig. 4a). Meanwhile, the overexpression of anti-miR-34a, but not anti-miR-34a-mut, increased the luciferase activity of the WT reporter vector (Fig. 4b). Subsequent studies revealed that only the WT MALAT1 target site is recognized by miR-34a (Fig. 4d) and anti-miR-34a (Fig. 4e). Rescue experiments were conducted to determine whether the effect of MALAT1 is dependent on miR-34a. We observed that MALAT1 rescued the luciferase activity associated with c-Myc and Met in the presence of miR-34a (Fig. 4f, g). Furthermore, the overexpression of MALAT1 resulted in the increased enrichment of Ago2 on MALAT1, but substantially decreased the enrichment on c-Myc and Met (Fig. 4h). These data demonstrate that MALAT1 contains functional miR-34a-binding sites.

### In vivo confirmation that MALAT1 functions as a miR-34a sponge

**Fig. 5:**
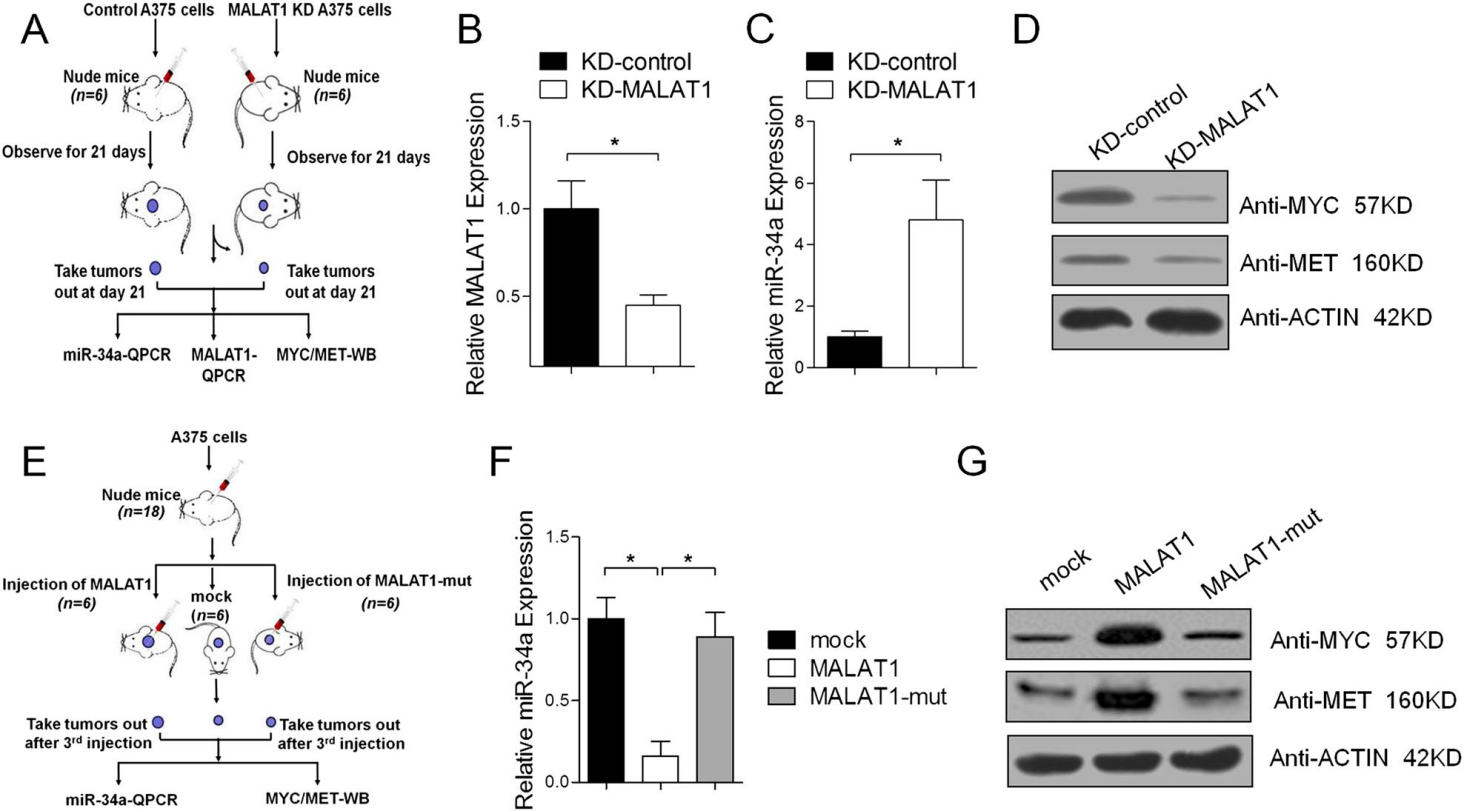
In vivo confirmation that MALAT1 functions as a miR-34a sponge. **a** Schematic diagram of the animal experimental design. The A375 cells stably expressing either sh-lncRNA-MALAT1 or sh-NC were injected subcutaneously into the dorsal flanks of the indicated numbers of BALB/c nude mice. (**b**–**c)** At 21 days after injection, the xenografts were dissected and examined in a quantitative real-time polymerase chain reaction (qRT-PCR) assay to analyze MALAT1 (**b**) and miR-34a (**c**) expression levels. **d** c-Myc and Met protein levels were analyzed in a western blot. **e** Schematic diagram of the animal experimental design. Stable A375 cells with pLVX-IRES-Puro-MALAT1 or pLVX-IRES-Puro-MALAT1-mut were injected subcutaneously into the dorsal flanks of 5-week-old male BALB/c nude mice. **f** Xenografts were dissected 21 days after injections, and MALAT1 and MALAT1-mut expression levels were analyzed in a qRT-PCR assay. **g** c-Myc and Met protein levels were analyzed in a western blot

To determine whether MALAT1 functions as a molecular sponge for miR-34a in vivo, qRT-PCR and western blot experiments were completed to analyze the expression of MALAT1 and miR-34a in mice. Compared with the negative control, the relative expression of miR-34a was higher in the MALAT1 knockdown xenograft (Fig. 5c). The western blot indicated that the c-Myc and Met protein levels decreased when MALAT1 was knocked down (Fig. 5d). Conversely, MALAT1 overexpression decreased miR-34a expression, but not when the miR-34a-binding site of MALAT1 was mutated (Fig. 5f). Moreover, the overexpression of MALAT1 led to increased c-Myc and Met levels, but not if the miR-34a-binding site of MALAT1 was mutated (Fig. 5g). These results suggested that MALAT1 functions as a molecular sponge for miR-34a in vivo.

### The expression of miR-34a is inversely associated with MALAT1 in melanoma tissues

**Fig. 6:**
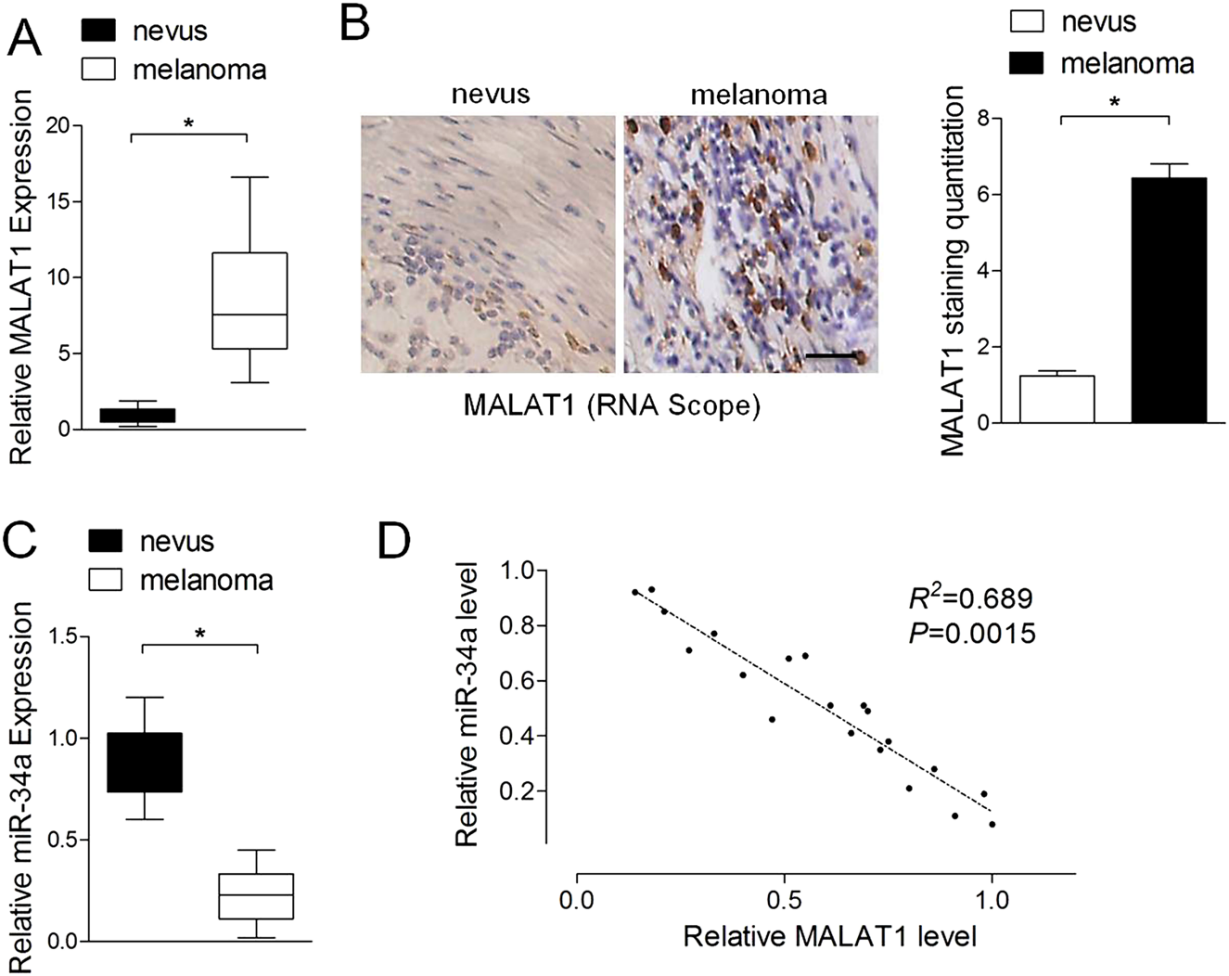
The expression of miR-34a is inversely associated with MALAT1 in melanoma tissues. **a** Relative MALAT1 expression levels in melanoma tissues (*n* = 20) and benign nevi (*n* = 20) were analyzed in a quantitative real-time polymerase chain reaction (qRT-PCR) assay. **b** RNAscope detection of MALAT1 expression in melanoma tissues (*n* = 20) and benign nevi (*n* = 20). Left panel: representative images. Scale bars: 100 µm. Right panel: data analysis. **c** Relative miR-34a expression levels in melanoma tissues (*n* = 20) and benign nevi (*n* = 20) were analyzed in a qRT-PCR assay. **d** miR-34a expression is inversely associated with MALAT1 in melanoma tissues (*r*^2^ = 0.689, *P* = 0.0015)

To explore whether MALAT1 regulates miR-34a in clinical tissue samples, we analyzed miR-34a expression and assessed its correlation with MALAT1 in 20 melanoma tissues and 20 nevi. The qRT-PCR results showed that MALAT1 was more highly expressed in the melanoma tissues than in the benign nevi (Fig. 6a). The results of the RNAscope assay were consistent with those of the qRT-PCR, with significantly higher MALAT1 levels in melanoma tissues than in benign nevi (Fig. 6b). In contrast, the miR-34a expression level was lower in melanoma tissues than in benign nevi (Fig. 6c). Pearson’s correlation analyses revealed that miR-34a expression was inversely associated with MALAT1 in melanoma tissues (*r*^2^ = 0.689, *P* = 0.0015) (Fig. 6d). These data suggest that MALAT1 regulates miR-34a in melanoma tissues.

## Discussion

Melanoma is the deadliest form of skin cancer, and there are limited options for treating advanced melanoma^51^. Hence, identifying novel targets for melanoma therapy is critical. In this study, we first determined that miR-34a is strongly and negatively regulated by MALAT1 based on experiments in which the lncRNA was overexpressed. Previous studies revealed that MALAT1 functions as a molecular sponge to inhibit miRNA expression, and plays a significant role in cancer pathogenesis^52–55^. This study presents strong evidence that MALAT1 is overexpressed in melanoma tissues and functions as a molecular sponge that competitively inhibits miR-34a and modulates the abundance of the targeted c-Myc and Met. The expression of miR-34a, which is a member of the miR-34 family, is downregulated in neuroblastoma, pancreatic cancer, prostate cancer, lung cancer, malignant lymphoma, retinoblastoma, and colon cancer^22,56–59^. In the present study, we demonstrated that the miR-34a expression level is lower in melanoma tissues than in benign nevi.

Earlier investigations concluded that MALAT1 expression is upregulated in many tumors, including those of bladder cancer, ovarian cancer, gastric cancer, osteosarcoma, and pancreatic cancer^60–64^. Moreover, MALAT1 is considered to be a proto-oncogene in an increasing number of human tumor tissues^65–68^. In this study, we revealed that MALAT1 is more highly expressed in melanoma tissues than in benign nevi, and it contains functional sequence-specific miR-34a-binding sites. Knocking down MALAT1 significantly upregulated the expression of miR-34a.

Luciferase reporter analyses with the co-transfection of miR-34a, a MALAT1 expression plasmid, and c-Myc or a Met luciferase reporter vector clearly indicated that miR-34a suppresses c-Myc and Met luciferase activity and that the miR-34a function is mediated by MALAT1. In addition, miR-34a significantly repressed WT MALAT1, but a mutated putative MALAT1-binding site (Fig. 4) did not completely abolish the repression by miR-34a (Fig. 4), suggesting that miR-34a may also bind to other sequences in MALAT1. Knocking down MALAT1 suppressed the transcription of *Met* (Fig. 3g), but not *c-Myc* (Fig. 3h). This result may be due to the following: 1) miRNAs bind to the 3ʹ-UTR of mRNAs to inhibit protein production. Perhaps only *Met* expression was inhibited by miR-34a in A375 cells; 2) Gene regulation is very complex, especially in various cancer cell lines. Consequently, *c-Myc* mRNA may have been negatively affected by miR-34a in other cells, but not in A375 cells. In fact, our results are consistent with those from other studies. For example, Christoffersen et al. observed that miR-34a targets *MYC* during B-RAF-induced senescence, but the overexpression of a miR-34a precursor in TIG3 TERT/DB-RAF:ER cells has little effect on *MYC* expression at the mRNA level^69^. Disayabutr et al. also reported that miR-34 targets *c-Myc* mRNA in type II alveolar epithelial cells, but the overexpression of miR-34a does not markedly influence *c-Myc* transcription in lung epithelial cells^70^. We also searched in TargetScan and found there were two well-conserved miR-34-binding sites (position 51–67 bp and 2165-2171 bp) in Met 3ʹ-UTR but not found in c-Myc 3ʹ-UTR.

In summary, we demonstrated that miR-34a expression is inversely associated with MALAT1 in melanoma tissues. Moreover, MALAT1 functions as a molecular sponge for miR-34a, and helps regulate the expression of *c-Myc* and *Met* in melanoma cells. We also proved that miR-34a is targeted by MALAT1. Future investigations of the crosstalk between MALAT1 and miR-34a may provide useful insights for developing new strategies to improve the efficacy of melanoma treatments.
